# Pharmacokinetics, Safety, and Tolerability of Single and Multiple Doses of Relebactam, a β-Lactamase Inhibitor, in Combination with Imipenem and Cilastatin in Healthy Participants

**DOI:** 10.1128/AAC.00280-18

**Published:** 2018-08-27

**Authors:** Elizabeth G. Rhee, Matthew L. Rizk, Nicole Calder, Marcela Nefliu, Steven J. Warrington, Michael S. Schwartz, Eric Mangin, Keith Boundy, Pratik Bhagunde, Francheska Colon-Gonzalez, Patricia Jumes, Yang Liu, Joan R. Butterton

**Affiliations:** a Merck & Co., Inc., Kenilworth, New Jersey, USA; b Hammersmith Medicines Research Ltd., London, United Kingdom

**Keywords:** pharmacokinetics, relebactam, β-lactamase inhibitor

## Abstract

Relebactam is a novel class A and C β-lactamase inhibitor that is being developed in combination with imipenem-cilastatin for the treatment of serious infections with Gram-negative bacteria. Here we report on two phase 1 randomized, double-blind, placebo-controlled pharmacokinetics, safety, and tolerability studies of relebactam administered with or without imipenem-cilastatin to healthy participants: (i) a single-dose (25 to 1,150 mg) and multiple-dose (50 to 625 mg every 6 h [q6h] for 7 to 14 days) escalation study with men and (ii) a single-dose (125 mg) study with women and elderly individuals. Following single- or multiple-dose intravenous administration over 30 min, plasma relebactam concentrations declined biexponentially, with a terminal half-life (*t*_1/2_) ranging from 1.35 to 1.85 h independently of the dose. Exposures increased in a dose-proportional manner across the dose range. No clinically significant differences in pharmacokinetics between men and women, or between adult and elderly participants, were observed. Urine pharmacokinetics demonstrated that urinary excretion is the major route of relebactam elimination. No drug-drug interaction between relebactam and imipenem-cilastatin was observed, and the observed *t*_1/2_ values for relebactam, imipenem, and cilastatin were comparable, thus supporting coadministration. Relebactam administered alone or in combination with imipenem-cilastatin was well tolerated across the dose ranges studied. No serious adverse events or deaths were reported. The pharmacokinetic profile and favorable safety results supported q6h dosing of relebactam with imipenem-cilastatin in clinical treatment trials.

## Abstract (precis)

Relebactam is a novel class A and C β-lactamase inhibitor that is being developed in combination with imipenem-cilastatin for the treatment of serious infections with Gram-negative bacteria. Here we report on two phase 1 randomized, double-blind, placebo-controlled pharmacokinetics, safety, and tolerability studies of relebactam administered with or without imipenem-cilastatin to healthy participants: (i) a single-dose (25 to 1,150 mg) and multiple-dose (50 to 625 mg every 6 h [q6h] for 7 to 14 days) escalation study with men and (ii) a single-dose (125 mg) study with women and elderly individuals.

## INTRODUCTION

Gram-negative bacteria are a common cause of serious infections, including intra-abdominal infections (IAIs), urinary tract infections (UTIs), nosocomial pneumonia, and bacteremia (1–4). Antibiotic-resistant bacteria are considered a global health threat by the Centers for Disease Control and Prevention and the World Health Organization (5–7) and are associated with increased mortality, hospital readmissions, and costs (8–10). The emergence of new β-lactamases, including carbapenemases such as Klebsiella pneumoniae carbapenemase (KPC), has rendered standard β-lactam antibiotics for Gram-negative infections less effective (5, 10–13). In an effort to overcome antibiotic resistance, new β-lactam–β-lactamase inhibitor combinations are being tested, and others have been approved. Ceftolozane-tazobactam and ceftazidime-avibactam combinations were approved for the treatment of both complicated IAIs and UTIs in 2014 and 2015, respectively (14–17).

Carbapenems are an effective treatment against many resistant Gram-negative bacteria, retaining activity against many extended-spectrum β-lactamase producers (18). Imipenem, a broad-spectrum carbapenem antibiotic that is coformulated with cilastatin, was approved by the U.S. Food and Drug Administration (FDA) in 1985 for the treatment of complicated UTIs, pneumonia, and other serious bacterial infections (19–22). Cilastatin, a dehydropeptidase inhibitor, is used in combination with imipenem to prevent its degradation by renal dehydropeptidase I and to promote efficacy (23). However, resistance to imipenem-cilastatin has increased over the past 2 decades; it is now estimated that the rate of infection with carbapenem-resistant bacteria is increasing, with a 5-fold increase reported between 2008 and 2012 in community hospitals, emphasizing the need for novel agents (24, 25).

Relebactam (MK-7655) is an inhibitor of class A and class C β-lactamases that is in development for use in combination with imipenem-cilastatin to treat serious infections caused by β-lactamase-producing Gram-negative bacteria (26, 27). The addition of relebactam restores imipenem activity against multidrug-resistant strains of Pseudomonas aeruginosa and Enterobacteriaceae *in vitro* (26, 28). Relebactam decreased imipenem MICs from a range of 16 to 64 mg/liter to a range of 0.12 to 1 mg/liter for Enterobacteriaceae with KPC. For P. aeruginosa, relebactam decreased imipenem MICs from a range of 1 to 2 mg/liter to a range of 0.25 to 0.5 mg/liter for imipenem-susceptible strains and from a range of 16 to 64 mg/liter to a range of 1 to 4 mg/liter for OprD-deficient strains (28). In phase 2 clinical studies, the combination of relebactam with imipenem-cilastatin for the treatment of complicated UTIs and IAIs has been well tolerated, with a safety profile similar to that of imipenem-cilastatin alone (29, 30).

**FIG 1:**
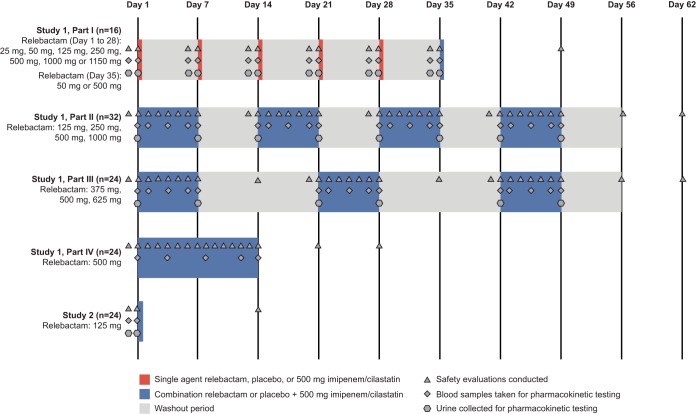
Study design. Predose baseline safety evaluations were conducted, and blood and urine samples were collected within 24 h prior to dosing. Safety assessments were conducted through the duration of dosing and continued through approximately 14 days after the last dose.

Here we present the results of two phase 1 studies that investigated the pharmacokinetics, safety, and tolerability of relebactam alone and in combination with imipenem-cilastatin for healthy adult men (study 1), and for healthy adult women, elderly men, and elderly women (study 2) (Fig. 1).

## RESULTS

### Participants.

A total of 106 and 24 participants were enrolled in study 1 (protocol 001) and study 2 (protocol 002), respectively. Study 1 panels A through K comprised men, mostly Caucasian, with median ages between 24.5 and 31.5 years (Table 1). All participants in study 2 were Hispanic; panel L comprised elderly men, mostly Caucasian, with a median age of 68 years; panel M comprised elderly women, all Caucasian, with a median age of 61 years; and panel N comprised adult women, all Caucasian, with a median age of 31 years (Table 1).

**TABLE 1:** Demographic and baseline characteristics in studies 1 and 2

Study and panel	No. of participants	Age (yr)		No. (%) with the following characteristic:								
				Gender		Ethnicity		Race				
		Mean (SD)	Median (range)	Male	Female	Not Hispanic or Latino	Hispanic or Latino	Caucasian	Black or African-American	Asian	Multiracial	Unknown
1												
A	8	30.8 (8.7)	31.5 (19–40)	8 (100)	0	8 (100)	0	5 (62.5)	2 (25.0)	1 (12.5)	0	0
B	8	29.3 (7.8)	28.5 (18–40)	8 (100)	0	8 (100)	0	4 (50)	1 (12.5)	3 (37.5)	0	0
C	8	27.9 (7.8)	25.0 (20–45)	8 (100)	0	8 (100)	0	8 (100)	0	0	0	0
D	8	26.4 (2.5)	25.5 (24–31)	8 (100)	0	7 (87.5)	1 (12.5)	4 (50)	2 (25)	1 (12.5)	0	1 (12.5)
E	8	30.0 (4.7)	28.5 (24–38)	8 (100)	0	8 (100)	0	7 (87.5)	0	1 (12.5)	0	0
F	9	27.7 (6.5)	27.0 (19–39)	9 (100)	0	8 (100)	0	7 (77.8)	1 (11.1)	0	1 (11.1)	0
G	8	27.1 (6.4)	25.5 (20–38)	8 (100)	0	8 (100)	0	8 (100)	0	0	0	0
H	8	26.4 (5.0)	24.5 (21–35)	8 (100)	0	8 (100)	0	7 (87.5)	0	1 (12.5)	0	0
I	9	27.9 (6.1)	27.0 (20–37)	9 (100)	0	9 (100)	0	9 (100)	0	0	0	0
J	16	27.1 (6.2)	26.0 (20–41)	16 (100)	0	9 (100)	0	13 (81.3)	3 (18.8)	0	0	0
K	16	27.4 (6.3)	26.0 (20–38)	16 (100)	0	8 (100)	0	16 (100)	0	0	0	0
2												
L	8	67.6 (4.9)	68.0 (61–75)	8 (100)	0	0	8 (100)	7 (87.5)	1 (12.5)	0	0	0
M	8	62.8 (5.0)	61.0 (60–75)	0	8 (100)	0	8 (100)	8 (100)	0	0	0	0
N	8	32.0 (5.2)	31.0 (26–39)	0	8 (100)	0	8 (100)	8 (100)	0	0	0	0

### Pharmacokinetics. (i) Plasma relebactam concentrations following single-dose administration.

**FIG 2:**
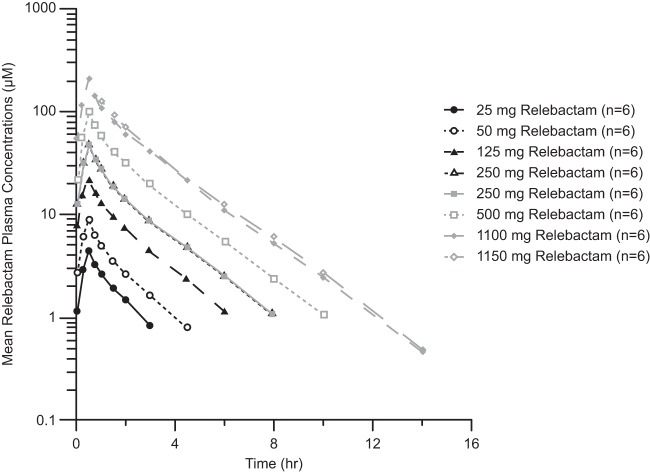
Plasma relebactam concentration-versus-time profiles (on a semilog scale) following administration of single doses (25 mg to 1,150 mg) of relebactam to healthy male participants in study 1. Concentrations were measured in 2 patient cohorts, indicated by black (panel A) or gray (panel B) lines. Within each cohort, participants received 250 mg relebactam during one of the study periods.

Following single-dose administration of relebactam as a 30-min infusion to healthy men (part I), plasma relebactam concentrations declined biexponentially, with a harmonic mean terminal half-life (*t*_1/2_) ranging from 1.35 to 1.8 h over the 25- to 1,150-mg dose range (Table 2). The observed *t*_1/2_ was comparable to that of imipenem (1.1 h), supporting the proposed q6h (every 6 h) dosing regimen for coadministration of these agents. The mean plasma exposures (area under the concentration-time curve from 0 h to infinity [AUC_0–∞_]) and maximum concentration of drug in serum (*C*_max_) increased with an approximately dose-proportional relationship over the 25- to 1,150-mg dose range of relebactam (Fig. 2). The target AUC_0–∞_ of 37.5 μM · h (13.1 mg · h/liter) was attained at doses of 125 mg and higher when relebactam was administered as a single agent.

**TABLE 2:** Plasma pharmacokinetic parameters following administration of a single dose of relebactam (25 to 1,150 mg) with or without 500 mg imipenem-cilastatin to healthy men in study 1

Drug	No. of participants	Dose (mg)		Value for the following parameter^*a*^:				
		Relebactam	Imipenem-cilastatin	*C*_EOI_ (μM)^*b*^	AUC_0–∞_ (μM · h)	*t*_1/2_ (h)	CL (ml/min)	*V_z_* (liters)
Relebactam	6	25	0	4.42 ± 1.18	7.97 ± 1.67	1.39 ± 0.246	155 ± 30.6	18.3 ± 2.99
	6	50	0	9.04 ± 1.88	15.6 ± 2.73	1.52 ± 0. 248	157 ± 23.8	20.5 ± 3.88
	6	125	0	21.9 ± 5.46	40.6 ± 4.41	1.58 ± 0.122	148 ± 14.8	20.5 ± 3.49
	12	250	0	45.4 ± 7.27	81.8 ± 9.07	1.63 ± 0.129	148 ± 16.4	20.8 ± 2.77
	6	500	0	101 ± 17.6	180 ± 25.7	1.64 ± 0.141	135 ± 19.9	19.1 ± 2.64
	6	1,000	0	209 ± 28.6	373 ± 51.9	1.84 ± 0.281	130 ± 18.3	20.6 ± 3.46
	6	1,150	0	208 ± 18.3	396 ± 55.3	1.78 ± 0.225	141 ± 18.8	21.6 ± 2.69
	6	50	500	9.13 ± 2.06	16.4 ± 1.99	1.58 ± 0.384	148 ± 15.8	20.1 ± 4.79
	6	500	500	93.2 ± 10.5	183 ± 25.9	1.75 ± 0.318	133 ± 19.4	19.9 ± 3.59
Imipenem	8	0	500	102 ± 17.0	140 ± 16.7	1.13 ± 0.075	201 ± 21.4	19.6 ± 2.43
	8	0	500	105 ± 12.3	142 ± 19.1	1.16 ± 0.110	200 ± 27.2	19.8 ± 1.84
	6	50	500	105 ± 22.7	138 ± 17.1	1.10 ± 0.136	204 ± 21.3	19.5 ± 3.66
	6	500	500	103 ± 10.1	149 ± 20.1	1.16 ± 0.156	189 ± 24.4	18.9 ± 3.19
Cilastatin	8	0	500	101 ± 16.6	114 ± 13.5	0.920 ± 0.051	207 ± 23.1	16.5 ± 2.11
	8	0	500	113 ± 14.4	132 ± 26.7	1.00 ± 0.113	183 ± 39.0	15.6 ± 2.14
	6	50	500	104 ± 22.9	113 ± 18.2	0.919 ± 0.159	210 ± 32.9	16.8 ± 4.48
	6	500	500	101 ± 12.2	125 ± 23.9	0.969 ± 0.162	194 ± 44.1	15.9 ± 2.74

a *C*_EOI_, concentration at end of infusion; AUC_0–∞_, area under the concentration-time curve from 0 h to infinity; *t*_1/2_, apparent terminal half-life; CL, clearance; *V_z_*, apparent volume of distribution at terminal phase.

b Concentrations for this study are expressed as μM; to convert to mg · h/liter, multiply by 0.34848 for relebactam and 0.29937 for imipenem.

### (ii) Combination of relebactam with imipenem-cilastatin.

**FIG 3:**
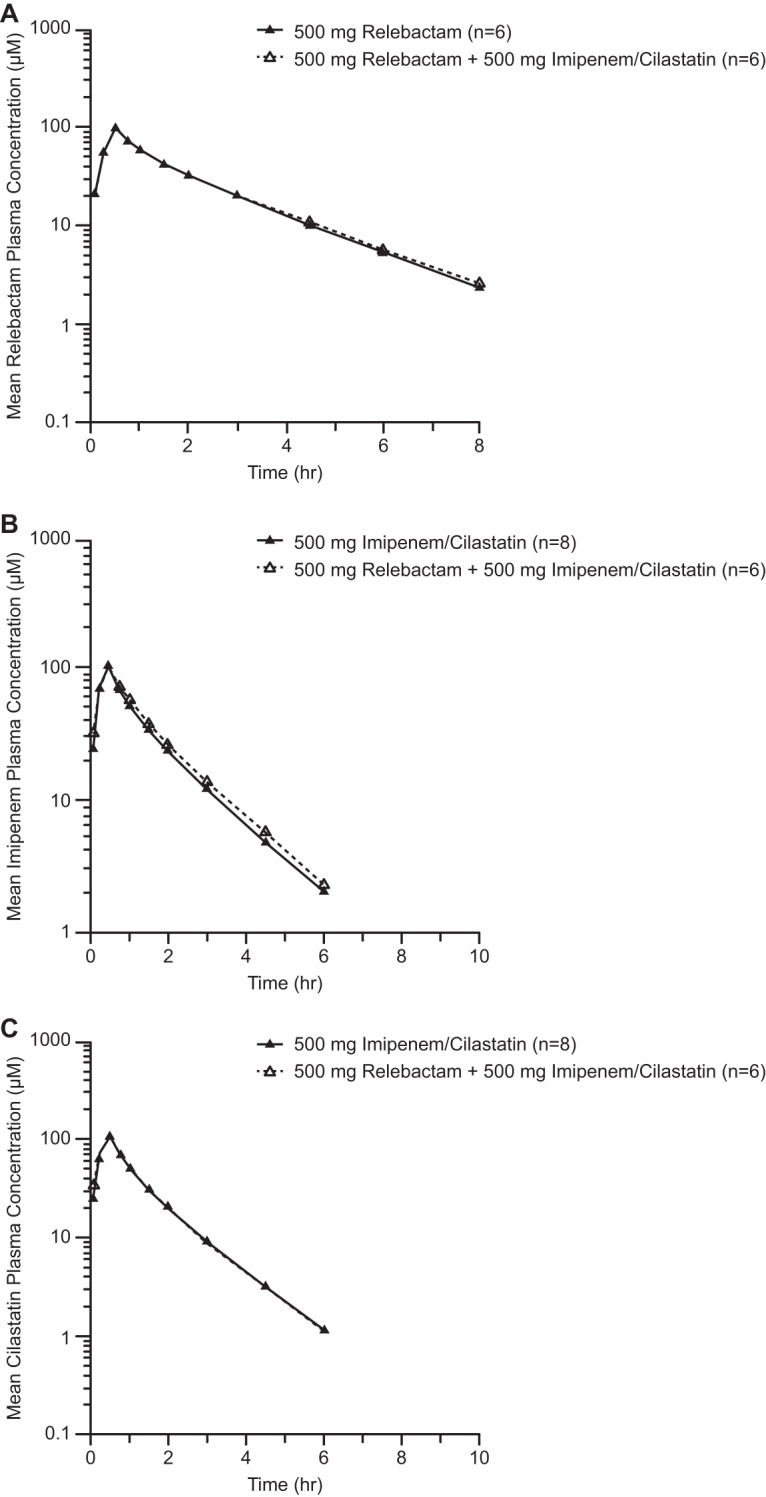
Arithmetic mean concentrations of relebactam (A), imipenem (B), and cilastatin (C) in plasma (on a semilog scale) over time following administration of a single 500-mg i.v. dose of relebactam, either alone or together with imipenem-cilastatin, to healthy male patients in study 1.

The pharmacokinetics of relebactam were similar when it was dosed with and without 500 mg of imipenem-cilastatin, and no evidence of a drug-drug interaction was detected (Fig. 3). The geometric mean ratios (GMRs [90% confidence intervals {CIs}]) for AUC_0–∞_ were 1.02 (0.97, 1.06) for relebactam, 1.03 (0.95, 1.12) for imipenem, and 0.91 (0.87, 0.95) for cilastatin. The GMRs (90% CIs) for the concentration at end of infusion (*C*_EOI_) were 0.93 (0.85, 1.02) for relebactam, 0.98 (0.89, 1.08) for imipenem, and 0.87 (0.81, 0.92) for cilastatin. In addition, the pharmacokinetics of imipenem and cilastatin were similar when imipenem-cilastatin was dosed with and without relebactam in that all data fell within standard bioequivalence bounds (0.80 to 1.25).

### (iii) Effects of gender and age.

**FIG 4:**
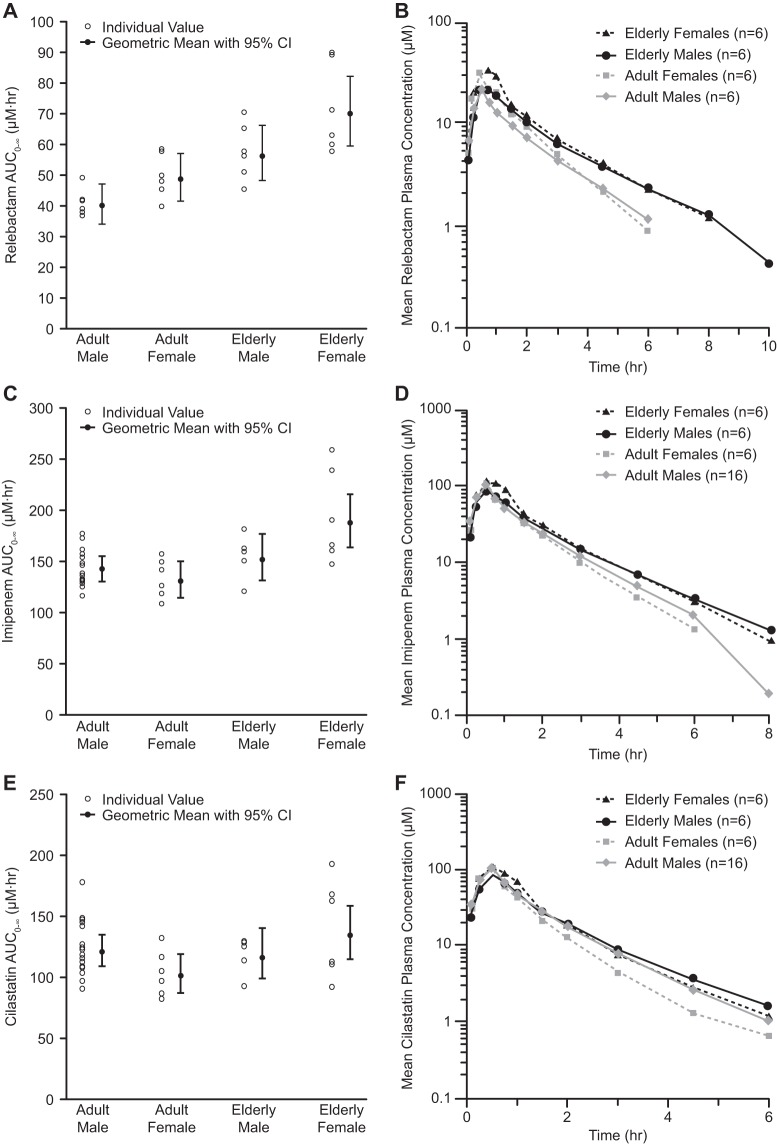
Plasma exposures and concentration-versus-time profiles following administration of a single dose of relebactam with imipenem-cilastatin. Shown are plasma exposures of relebactam (A), imipenem (C), and cilastatin (E) and plasma concentration-versus-time profiles for relebactam (B), imipenem (D), and cilastatin (F) on a semilog scale.

Following single-dose administration of relebactam to elderly men, elderly women, and adult women, AUC_0–∞_ values for relebactam were slightly higher in both elderly and adult woman populations than in males of the associated age group (historic data from study 1, panel C), increasing by 25% and 22%, respectively (Table 3; Fig. 4). Elderly men and women had higher mean plasma relebactam exposures than adult counterparts of the same gender, increasing by 41% and 45%, respectively. The mean relebactam *C*_EOI_ was higher in both adult and elderly women than in men in the corresponding age groups. Elderly men and women had numerically higher *C*_EOI_ values than adult participants of the same gender. The mean plasma clearance (CL) and apparent volume of distribution during terminal phase (*V_z_*) were slightly lower in both adult and elderly women than in men of the same age groups. Lower CL was observed in both elderly men and women than in adult participants. The relebactam *V_z_* was similar among age groups, and apparent *t*_1/2_ was also similar in the gender and age comparisons. Overall, age and gender did not have a clinically relevant effect on the pharmacokinetic parameters of relebactam. Imipenem and cilastatin mean exposures, AUC_0–∞_, *C*_EOI_, CL, *V_z_*, and apparent *t*_1/2_ were comparable between genders and between elderly and adult participants.

**TABLE 3:** Plasma pharmacokinetic parameters following administration of a single dose of 125 mg relebactam with 500 mg imipenem-cilastatin in study 2

Drug and group	No. of participants	Value (mean ± SD) for the following parameters^*a*^:				
		*C*_EOI_ (μM)^*b*^	AUC_0–∞_ (μM · h)	*t*_1/2_ (h)	CL (ml/min)	*V_z_* (liters)
Relebactam						
Adults						
Men^*c*^	6	21.9 ± 5.46	40.6 ± 4.41	1.58 ± 0.122	148 ± 14.8	20.5 ± 3.49
Women	6	30.5 ± 5.05	49.5 ± 7.29	1.28 ± 0.099	123 ± 18.5	13.5 ± 1.46
Elderly						
Men	6	22.9 ± 4.47	57.4 ± 9.24	2.21 ± 0.382	106 ± 17.4	20.1 ± 3.49
Women	6	37.3 ± 11.5	71.6 ± 14.6	1.92 ± 0.301	86.3 ± 16.5	14.0 ± 0.682
Imipenem						
Adults						
Men^*d*^	16	102 ± 17.0	140 ± 16.7	1.13 ± 0.075	201 ± 21.4	19.6 ± 2.43
Women	6	105 ± 12.8	132 ± 18.9	0.977 ± 0.0535	215 ± 31.5	18.1 ± 1.81
Elderly						
Men	5	85.8 ± 18.2	153 ± 22.3	1.46 ± 0.185	185 ± 29.8	23.2 ± 2.77
Women	6	128 ± 36.4	192 ± 45.4	1.31 ± 0.225	152 ± 33.3	16.6 ± 1.06
Cilastatin						
Adults						
Men^*d*^	16	101 ± 16.6	114 ± 13.5	0.920 ± 0.0507	207 ± 23.1	16.5 ± 2.11
Women	6	102 ± 10.9	102 ± 18.6	0.832 ± 0.112	234 ± 41.4	16.5 ± 1.45
Elderly						
Men	5	83.9 ± 12.5	118 ± 15.7	1.23 ± 0.189	201 ± 30.6	21.4 ± 5.03
Women	6	119 ± 34.3	139 ± 39.7	1.16 ± 0.255	180 ± 52.4	17.2 ± 2.38

a *C*_EOI_, concentration at end of infusion; AUC_0–∞_, area under the concentration-time curve from 0 h to infinity; CL, clearance; *t*_1/2_, half-life; *V_z_*, apparent volume of distribution at terminal phase.

b Concentrations for this study are expressed in μM; to convert to mg · h/liter, multiply by 0.34848 for relebactam and 0.29937 for imipenem.

c Participants from study 1 given a 125-mg dose of relebactam.

d Participants from study 1 given a 500-mg dose of imipenem-cilastatin.

### (iv) Urinary pharmacokinetics.

Relebactam was almost completely excreted in urine, with the fraction excreted (fe) ranging from 94.7% to 100% over a 24-h period following single-dose administration to healthy men (see Table S1 in the supplemental material). The renal clearance (CL_R_) and fe were similar across the entire dose range (25 to 1,150 mg) and when relebactam was administered with and without imipenem-cilastatin.

For fe and CL_R_ values, numerical differences were observed between groups with overlapping 95% CIs (Table 4). In gender- and age-based comparisons, the mean CL_R_ was lower for women than for men with relebactam (106 versus 158 ml/min), imipenem (98.6 versus 108 ml/min), and cilastatin (157 versus 168 ml/min). Similarly, CL_R_ was numerically lower for elderly participants than for adult participants with relebactam (69.6 to 89.2 versus 106 to 158 ml/min), imipenem (67.6 to 80.9 versus 98.6 to 108 ml/min), and cilastatin (112 to 126 versus 157 to 168 ml/min). The mean fe was not significantly different between elderly men and women for any of the agents, nor was it different between elderly and adult women; however, mean fe was numerically lower in adult women than in adult men, as well as in elderly men than in adult men, for all agents. The minor differences in fe and CL_R_ between gender and age groups did not have a clinically relevant effect on the urine pharmacokinetics of relebactam, imipenem, or cilastatin.

**TABLE 4:** Summary of urine pharmacokinetic parameters following administration of a single dose of 125 mg relebactam with 500 mg imipenem-cilastatin in study 2

Drug and group	No. of participants	Mean value (95% CI) for the following parameters^*a*^:	
		CL_R_ (ml/min)	fe (%, least squares mean)
Relebactam			
Adults			
Men^*b*^	6	158 (128, 194)	103 (93.9, 111)
Women	6	106 (86.2, 131)	84.2 (75.4, 93.0)
Elderly			
Men	6	89.2 (72.4, 110)	80.7 (71.9, 89.5)
Women	6	69.6 (56.5, 85.7)	79.6 (70.8, 88.4)
Imipenem			
Adults			
Men^*c*^	16	108 (97.0, 120)	55 (50.6, 59.4)
Women	6	98.6 (82.7, 118)	45.8 (38.6, 53.0)
Elderly			
Men	5	80.9 (66.7, 98.0)	44.7^*d*^ (37.5, 51.9)
Women	6	67.6 (56.7, 80.6)	45.1 (37.9, 52.3)
Cilastatin			
Adults			
Men^*c*^	16	168 (148, 191)	88.5 (82.1, 94.9)
Women	6	157 (127, 194)	68.2 (57.7, 78.6)
Elderly			
Men	5	126 (100, 159)	62.2^*d*^ (51.7, 72.6)
Women	6	112 (90.3, 138)	63.8 (53.3, 74.3)

a CI, confidence interval; CL_R_, renal clearance; fe, fraction excreted.

b Participants from study 1 given a 125-mg dose of relebactam.

c Participants from study 1 given a 500-mg dose of imipenem-cilastatin.

d *n* = 6.

### (v) Plasma relebactam concentrations following the administration of multiple doses.

Following the administration of relebactam q6h for 7 days (parts II and III), plasma concentrations declined biexponentially, with a geometric mean terminal *t*_1/2_ ranging from 1.42 to 1.85 h over the 50- to 625-mg dose range (see Table S2 in the supplemental material). The terminal *t*_1/2_ was similar between day 1 and day 7. An exploratory analysis of dose proportionality over the 50- to 625-mg relebactam dosing range suggested that AUC_0–6_ and *C*_EOI_ increased in an approximately dose proportional manner. When 500 mg relebactam was coadministered with imipenem-cilastatin q6h for 14 days (part IV), the pharmacokinetics, including terminal *t*_1/2_, were similar on day 1 and day 14. No accumulation of relebactam was observed in the 7- or 14-day periods following administration. The target AUC_0–6_ of 37.5 μM · h (13.1 mg · h/liter) was attained at relebactam doses of 125 mg and higher when relebactam was administered as a 30-min intravenous (i.v.) infusion q6h for 7 days. After multiple dosing of a proposed clinical dose of 250 mg relebactam, the average unbound concentration was 3.75 mg/liter.

### Safety. (i) Adverse events.

In study 1, no serious adverse events (AEs) or deaths were reported in any cohort, and no discontinuations due to AEs or clinically significant abnormalities in routine blood and urine chemistry were reported in the single-dose administration cohort (part I; *n* = 16). Eight of the 16 participants (50%) reported ≥1 drug-related AEs, the most common being somnolence, headache, and paresthesia (see Table S3 in the supplemental material). No trends were observed between AE incidence and increasing dose levels of relebactam.

In the multiple-dose administration panels (part II to part IV; *n* = 90), no serious AEs or deaths were reported. A total of 7 participants discontinued due to AEs, including 2 in the group receiving a placebo plus imipenem-cilastatin and 5 who received relebactam plus imipenem-cilastatin. In part II, a total of 3 participants receiving relebactam plus imipenem-cilastatin discontinued participation in the study for the following reasons: vomiting (*n* = 1; dose, 50 mg), nighttime dosing (*n* = 1; dose, 125 mg), and presyncope (*n* = 1; dose, 250 mg). In part III, 1 participant receiving a placebo with imipenem-cilastatin discontinued because of catheter site pain. In part IV, 2 discontinuations occurred because of rashes: 1 in a participant receiving a placebo and 1 in a participant receiving relebactam (500 mg) in combination with imipenem-cilastatin. One participant discontinued due to transaminase elevations. Overall, 66 of 90 participants (73%) reported at least 1 drug-related AE. These included infusion site erythema, infusion site pain, nausea, headache, catheter site pain, infusion site swelling, and tongue discoloration (Table 5).

**TABLE 5:** Study 1 participants (healthy men) with specific drug-related adverse events following multiple-dose administration of relebactam with 500 mg imipenem-cilastatin

Drug-related AE^*a*^	No. (%) for group receiving the indicated dose (mg) of relebactam plus 500 mg imipenem-cilastatin for the following period:									Total no. (%)^*b*^ (*n* = 90)
	7 days							14 days		
	50 (*n* = 6)	125 (*n* = 12)	250 (*n* = 7)	375 (*n* = 6)	500 (*n* = 6)	625 (*n* = 6)	0 (*n* = 15)	500 (*n* = 24)	0 (*n* = 8)	
Ear and labyrinth disorders (ear pain)	0	1 (8.3)	0	0	0	0	0	0	0	1 (1.1)
Eye disorders (eye pain)	0	1 (8.3)	0	0	0	0	0	0	0	1 (1.1)
Gastrointestinal disorders	1 (16.7)	3 (25.0)	1 (14.3)	1 (16.7)	3 (50.0)	1 (16.7)	1 (6.7)	10 (41.7)	3 (37.5)	24 (26.7)
Abdominal distension	1 (16.7)	1 (8.3)	0	0	0	0	0	0	0	2 (2.2)
Upper abdominal pain	0	0	0	0	0	1 (16.7)	0	0	0	1 (1.1)
Diarrhea	1 (16.7)	1 (8.3)	0	0	0	1 (16.7)	0	2 (8.3)	0	5 (5.6)
Dry mouth	0	1 (8.3)	0	0	0	0	0	0	0	1 (1.1)
Flatulence	0	0	0	0	1 (16.7)	0	0	0	0	1 (1.1)
Nausea	0	1 (8.3)	1 (14.3)	1 (16.7)	2 (33.3)	0	0	0	0	5 (5.6)
Salivary hypersecretion	0	1 (8.3)	0	0	0	0	0	0	0	1 (1.1)
Tongue discoloration	0	0	0	0	0	0	1 (6.7)	8 (33.3)	2 (25)	11 (12.2)
Tooth discoloration	0	0	0	0	0	0	0	1 (4.2)	1 (12.5)	2 (2.2)
Vomiting	1 (16.7)	0	0	0	0	0	0	0	0	1 (1.1)
General disorders and administration site conditions	3 (50.0)	9 (75.0)	3 (42.9)	5 (83.3)	3 (50.0)	3 (50.0)	8 (53.3)	11 (45.8)	5 (62.5)	50 (55.6)
Asthenia	0	0	0	0	0	0	0	1 (4.2)	0	1 (1.1)
Catheter site erythema	0	0	0	1 (16.7)	0	0	1 (6.7)	0	0	2 (2.2)
Catheter site pain	0	0	0	3 (50.0)	0	0	1 (6.7)	0	0	4 (4.4)
Catheter site swelling	0	0	0	1 (16.7)	0	0	0	0	0	1 (1.1)
Feeling hot	0	1 (8.3)	0	0	0	0	0	1 (4.2)	0	2 (2.2)
Feeling of body temp change	0	0	0	0	0	0	0	1 (4.2)	0	1 (1.1)
Infusion site erythema	3 (50.0)	9 (75.0)	2 (28.6)	4 (66.7)	3 (50.0)	3 (50.0)	6 (40.0)	9 (37.5)	5 (62.5)	44 (48.9)
Infusion site pain	2 (33.3)	6 (50.0)	3 (42.9)	3 (50.0)	2 (33.3)	3 (50.0)	6 (40.0)	6 (25.0)	2 (25.0)	33 (36.7)
Infusion site pruritus	1 (16.7)	0	0	0	0	1 (16.7)	0	0	0	2 (2.2)
Infusion site swelling	0	0	2 (28.6)	1 (16.7)	0	1 (16.7)	1 (6.7)	5 (20.8)	1 (12.5)	11 (12.2)
Investigations	0	0	0	0	0	0	0	1 (4.2)	0	1 (1.1)
ALT increase	0	0	0	0	0	0	0	1 (4.2)	0	1 (1.1)
AST increase	0	0	0	0	0	0	0	1 (4.2)	0	1 (1.1)
Metabolism and nutrition disorders (decreased appetite)	0	0	0	0	0	0	0	1 (4.2)	0	1 (1.1)
Musculoskeletal and connective tissue disorders	0	0	0	1 (16.7)	0	0	0	2 (8.3)	0	3 (3.3)
Arthralgia	0	0	0	0	0	0	0	1 (4.2)	0	1 (1.1)
Muscle twitching	0	0	0	0	0	0	0	1 (4.2)	0	1 (1.1)
Myalgia	0	0	0	0	0	0	0	1 (4.2)	0	1 (1.1)
Pain in extremity	0	0	0	1 (16.7)	0	0	0	0	0	1 (1.1)
Nervous system disorders	0	3 (25.0)	0	0	0	1 (16.7)	3 (20.0)	3 (12.5)	0	10 (11.1)
Ageusia	0	0	0	0	0	0	1 (6.7)	0	0	1 (1.1)
Dizziness	0	0	0	0	0	1 (16.7)	0	0	0	1 (1.1)
Dysgeusia	0	1 (8.3)	0	0	0	0	1 (6.7)	0	0	2 (2.2)
Headache	0	1 (8.3)	0	0	0	1 (16.7)	2 (13.3)	3 (12.5)	0	7 (7.8)
Lethargy	0	0	0	0	0	0	1 (6.7)	0	0	1 (1.1)
Somnolence	0	1 (8.3)	0	0	0	0	1 (6.7)	0	0	2 (2.2)
Respiratory, thoracic, and mediastinal disorders (oropharyngeal pain)	0	0	1 (14.3)	0	0	0	0	0	0	1 (1.1)
Skin and subcutaneous tissue disorders	1 (16.7)	0	0	2 (33.3)	0	0	1 (6.7)	1 (4.2)	1 (12.5)	6 (6.7)
Erythema	1 (16.7)	0	0	2 (33.3)	0	0	0	0	0	3 (3.3)
Hyperhidrosis	0	0	0	0	0	0	1 (6.7)	0	0	1 (1.1)
Rash	0	0	0	0	0	0	0	1 (4.2)	1 (12.5)	2 (2.2)

a AE, adverse event; ALT, alanine aminotransferase; AST, aspartate aminotransferase.

b Every participant was counted a single time for each AE category, so total values in the total AE categories may be less than the sum of the individual AEs.

In study 2, no serious AEs, laboratory AEs, events of clinical interest, study discontinuations, or deaths were reported. The majority of participants (*n* = 18 [75%]) did not experience any AE. The most common AE reported was vessel puncture site pain (8.3%). Two AEs were determined by the investigator to be drug related: 1 instance of dizziness reported in the group receiving relebactam with imipenem-cilastatin group, and 1 instance of a headache reported in the group receiving a placebo with imipenem-cilastatin. All drug-related AEs were transient and were considered mild or moderate by the investigator.

### (ii) Abnormal laboratory values.

Of the 42 participants who received multiple doses of relebactam with imipenem-cilastatin for 7 days (parts II and III) in study 1, 8 (19%) had transient elevated liver transaminases (alanine transaminase [ALT] and/or aspartate transaminase [AST]), up to 2.2-fold above the upper limit of normal (ULN) at 24 h after the last dose on day 7; this was observed in none of the 14 participants who received a placebo plus imipenem-cilastatin. In part IV, where dosing was extended to 14 days, a total of 6 of the 32 treated participants had elevated ALT values above the ULN while receiving either relebactam plus imipenem-cilastatin (4 participants) or a placebo plus imipenem-cilastatin (2 participants). The ALT elevations were <2-fold above the ULN, except for 1 participant in the group receiving relebactam plus imipenem-cilastatin who was withdrawn from the study on day 10 for an ALT value >3-fold the ULN. None of the liver function test elevations were associated with clinical findings; no clear dose effect was observed (elevations were observed with relebactam doses of 50 mg, 125 mg, 250 mg, 325 mg, 500 mg, and 625 mg); and elevations were reversible following the cessation of dosing. Overall, the incidence of ALT elevations above the ULN during 14 days of dosing was 25% (2/8) for subjects on a placebo plus imipenem-cilastatin and 15.7% (4/24) for subjects on relebactam plus imipenem-cilastatin.

## DISCUSSION

The results of these 2 phase 1 pharmacokinetics, safety, and tolerability studies that investigated the combination of relebactam plus imipenem-cilastatin in both adult and elderly participants supported further development of this β-lactam–β-lactamase inhibitor combination. In this analysis, relebactam concentrations declined biexponentially after peak concentrations, with a terminal elimination *t*_1/2_ in the range of 1.35 to 1.85 h independent of dose. Taken together with the observed *t*_1/2_ of imipenem, these findings support q6h dosing for coadministration of these agents. The analysis of the two-way drug-drug interaction between relebactam and imipenem or cilastatin showed no meaningful differences in pharmacokinetic parameters in the dosing regimens tested.

In the present analysis, after single and multiple dosing, the mean percentage of the administered dose of relebactam that was excreted unchanged in the urine ranged from 94.7% to 100% over a 24-h period for a single dose and from 89.2% to 99.5% over a 6-h period on day 7 for multiple doses. The mean observed CL_R_ was 135 ml/min on day 7 after multiple dosing of the 250-mg clinical dose of relebactam; these findings indicate that net active secretion of relebactam is involved in the renal elimination of relebactam and accounts for approximately 30% of the total clearance, since clearances exceeded the values expected due to filtration alone (unbound CL_R_ of 173 ml/min versus 120 ml/min, assuming a 78% free fraction for relebactam). The observed CL_R_ did not change significantly as a function of dose for either regimen (e.g., for relebactam doses of 50 mg, 250 mg, and 500 mg, the corresponding renal clearance rates were 141 ml/min, 135 ml/min, and 144 ml/min, respectively [Table S1 in the supplemental material]). Overall, the results show that urinary excretion is the major route of relebactam elimination. The pharmacokinetics of both imipenem and cilastatin were well characterized in the present analysis and were generally consistent with plasma and urine data reported previously for healthy adult and elderly participants (31–33).

Plasma exposure, plasma pharmacokinetics, and urine pharmacokinetics were generally similar for men and women, and for adult and elderly participants, indicating that dose modifications based on gender or age are not needed. Elderly and female participants had lower CL_R_ and fe for relebactam, imipenem, and cilastatin than men. Since all three analytes are primarily renally eliminated, the differences between age and gender groups may be attributed to a decrease in renal function associated with aging and to body weight differences between women and men.

For both study 1 and study 2, relebactam administered alone or in combination with imipenem-cilastatin was generally well tolerated in both healthy men and women, as well as in elderly and adult populations. No deaths or serious AEs occurred. Drug-related AEs were mild and similar in nature to those observed in phase 2 studies and described previously for imipenem-cilastatin alone (29, 30). The most common AEs were infusion site erythema (49%) and infusion site pain (37%) (Table 5). Infusion site reactions are common AEs associated with imipenem-cilastatin, although rates in the present studies were higher than those typically observed in imipenem-cilastatin studies, perhaps due to the inclusion of site reactions as a separate assessment in addition to routine AE collection (34). However, rates of infusion site erythema were comparable between treatment groups and ranged from 40.0% to 62.5% among participants receiving imipenem-cilastatin compared with 28.6 to 75.0% among those receiving imipenem-cilastatin with relebactam. Likewise, rates of infusion site pain were similar (25% to 40.0% versus 25% to 50% with relebactam).

Mild, asymptomatic hepatic transaminase elevations were noted with multiple-dose administration of relebactam or a placebo with imipenem-cilastatin. This laboratory finding was consistent with the AE profile of imipenem-cilastatin, which commonly causes mild and transient serum transaminase elevations, and resolved after the cessation of the study therapy (31, 34). No dose-related safety findings were noted for single doses of relebactam up to 1,150 mg and multiple doses coadministered with imipenem-cilastatin up to 625 mg q6h for 7 days. Overall, the addition of relebactam to imipenem-cilastatin in these phase 1 studies did not appreciably alter the imipenem-cilastatin safety profile.

The results from this study informed the doses of relebactam (125 and 250 mg) selected for evaluation in two studies that investigated relebactam with imipenem-cilastatin compared with imipenem-cilastatin alone in patients with complicated IAIs and complicated UTIs (29, 30). In the phase 2 studies, both doses met the pharmacokinetic target; there were no dose-related safety findings; and administration of 250 mg or 125 mg of relebactam with 500 mg imipenem-cilastatin administered q6h for 14 days was well tolerated. Population pharmacokinetic analysis from the complicated-IAI study (*n* = 351) demonstrated that the proposed dose of 250 mg relebactam in combination with 500 mg imipenem q6h provided coverage of >90% of carbapenem-resistant bacterial strains. Among patients with complicated IAIs caused by Escherichia coli, K. pneumoniae, and P. aeruginosa, coadministration of 250 or 125 mg of relebactam with imipenem-cilastatin resulted in favorable clinical responses in 96.3% or 98.8%, respectively (29). This combination was well tolerated, and the incidence of study discontinuation due to AEs was low (29). Among patients with complicated UTIs who were treated with 250 or 125 mg relebactam and imipenem-cilastatin, microbiological response rates were 95.5% or 98.6%, respectively (30). All patients (100%) with imipenem-resistant pathogens had favorable microbiological responses, demonstrating the effectiveness of this combination in this setting (30).

The selection of dosing regimens for subsequent phase 2 and phase 3 efficacy studies was supported by the pharmacokinetic and safety data provided in study 1 and study 2, and the optimal combination regimen (250 mg relebactam coadministered with 500 mg imipenem-cilastatin q6h) was further supported by part IV of study 1. The appropriateness of the dosing regimen was confirmed by a probability-of-target-attainment (PTA) analysis demonstrating >90% PTA following the initial phase 2 study for patients with complicated IAIs (29). This regimen has been evaluated in a phase 3 study of patients with imipenem-resistant bacterial infections (RESTORE-IMI 1 [ClinicalTrials registration no. NCT02452047]) and is being investigated in an ongoing phase 3 study of patients with hospital-acquired and ventilator-associated bacterial pneumonia (RESTORE-IMI 2 [ClinicalTrials registration no. NCT02493764]).

The increasing issue of antibiotic resistance creates a clear need for novel agents or antibiotic combinations with broad-spectrum efficacy and favorable safety profiles. Common Gram-negative bacteria, such as E. coli, K. pneumoniae, and P. aeruginosa, continue to develop resistance to standard antimicrobial agents (cephalosporins, carbapenems, and fluoroquinolones), and the incidence of resistance has increased over time in some species (1). Relebactam administered with imipenem-cilastatin combines a novel β-lactamase inhibitor with an established carbapenem regimen and has demonstrated robust *in vitro* and *in vivo* efficacy against imipenem-resistant strains.

## MATERIALS AND METHODS

### Study designs.

Study 1 (protocol 001) was a phase 1, randomized, double-blind, placebo-controlled, rising single- and multiple-dose study conducted at a single center that investigated relebactam in healthy adult men. The study was conducted in four parts, and all doses were administered intravenously (i.v.) in a total volume of 100 ml over 30 min.

Part I was an alternating two-panel (panels A and B) single-dose cohort of relebactam (25 to 1,150 mg) with or without 500 mg of imipenem-cilastatin (Fig. 1). Following an overnight fast, participants received either the active drug or a placebo in each of six periods with a minimum 7-day washout between doses. Panel A (*n* = 8) received the following escalating doses: (i) 25 mg relebactam, (ii) 50 mg relebactam, (iii) 125 mg relebactam, (iv) 500 mg imipenem-cilastatin, (v) 250 mg relebactam, and (vi) 50 mg relebactam plus 500 mg imipenem-cilastatin. Panel B (*n* = 8) received (i) 250 mg relebactam, (ii) 500 mg relebactam, (iii) 1,000 mg relebactam, (iv) 500 mg imipenem-cilastatin, (v) 1,150 mg relebactam, and (vi) 1,150 mg relebactam plus 500 mg imipenem-cilastatin. In each panel, in all periods (except period 4), subjects were randomized to receive a placebo (*n* = 2) or relebactam (*n* = 6). In period 4, all subjects received a single dose of 500 mg imipenem-cilastatin.

Parts II and III consisted of participants treated with 500 mg imipenem-cilastatin coadministered with a placebo or relebactam every 6 h (q6h) for 7 days. Each panel consisted of 8 participants, randomized to receive a placebo (*n* = 2) or relebactam (*n* = 6); different subjects participated in each panel. Each panel was dosed sequentially, allowing for assessment of safety and tolerability before dose escalation. Part II evaluated 50 mg (panel C), 125 mg (panel D), 125 mg (panel E), and 250 mg (panel F), and part III evaluated 375 mg (panel G), 500 mg (panel H), and 625 mg (panel I). In part IV, 32 participants were enrolled in two panels (panels J and K); participants were randomized to be coadministered multiple doses of 500 mg relebactam (*n* = 24) or a placebo (*n* = 8) and 500 mg imipenem-cilastatin q6h for 14 consecutive days.

Study 2 (protocol 002) was a phase 1, randomized, double-blind, placebo-controlled study with three panels of healthy participants that included elderly men (panel L [*n* = 8]), elderly women (panel M [*n* = 8]), and adult women (panel N [*n* = 8]). In each panel, subjects were randomized to the active drug (*n* = 6) or a placebo (*n* = 2). This study evaluated the pharmacokinetics, safety, and tolerability of 125 mg relebactam coadministered with 500 mg imipenem-cilastatin. Evaluations in this study included comparisons with healthy adult men (historic data [*n* = 99] from study 1) (Fig. 1).

These studies were conducted using standards established by the Declaration of Helsinki (study 2), the International Conference on Harmonization E6 good clinical practice guidelines (study 1 and study 2), and the 1997 UNESCO Declaration on the Human Genome and Human Rights (study 2) and were in compliance with all local and/or national regulations and directives (study 1 and study 2).

### Participants.

In study 1, eligible participants included heathy adult men (aged 18 to 45 years) who were nonsmokers and weighed ≥60 kg with a body mass index (BMI) of ≤30 kg/m^2^. Individuals with creatinine clearance (CL_CR_) of ≤80 ml/min were excluded. Participants eligible for study 2 included healthy elderly men aged 60 to 75 years (panel L), elderly women aged 60 to 75 years (panel M), and women aged 18 to 45 years (panel N) who were nonsmokers and weighed >55 kg with a BMI of ≤30 kg/m^2^. Participants with a CL_CR_ of ≤60 ml/min (panels L and M) or ≤80 ml/min (panel N) were excluded. In both studies, individuals who had a history of multiple and/or severe allergies or had experienced an anaphylactic reaction or significant intolerability to prescription or nonprescription drugs or food, as well as those with clinically significant neurological disease or cognitive impairment, or with endocrine, gastrointestinal, cardiovascular, hematological, hepatic, immunologic, renal, respiratory, or genitourinary diseases or neoplastic disease, were not eligible. In addition, individuals taking concomitant medications (prescription, nonprescription, or herbal remedies) who could not refrain from use beginning 2 weeks prior to the start of the study through the poststudy visit were excluded.

### Pharmacokinetic samples.

Blood samples were drawn into K_2_EDTA collection tubes for determination of relebactam, imipenem, and/or cilastatin concentrations before dosing and at 5, 15, 30, and 45 min and 1, 1.5, 2, 3, 4.5, 6, 8, 12, and 18 h after the start of the first i.v. drug infusion. The plasma was mixed with a stabilizer containing morpholineethanesulfonic acid buffer and ethylene glycol before storage at −70°C. Urine samples were either collected directly into collection bottles or immediately transferred from a void bottle before dosing and 0 to 3, 3 to 6, and 6 to 24 h postdosing. Aliquots were mixed with the stabilizer and stored frozen at −70°C until shipment to the research laboratories of Merck & Co., Inc., West Point, PA, USA, for the determination of relebactam, imipenem, and/or cilastatin concentrations. Plasma and urine relebactam, imipenem, and/or cilastatin concentrations were measured simultaneously using a validated method from the laboratories of Merck & Co., Inc., West Point, PA, USA. The assay utilized hydrophilic interaction chromatography (HILIC) and tandem mass spectrometry. Stable isotope-labeled internal standards were used for all three analytes. The per-protocol population included in this analysis comprised participants who received the active drug and complied with the protocol sufficiently to ensure that the data generated exhibited the effects of the active drug, according to the underlying scientific model.

### Safety assessments.

Participants were domiciled at the clinical research unit throughout the receipt of the study drug and for 24 h following administration. Safety and tolerability were monitored by clinical assessment of AEs and by repeated measurements of vital signs, physical examinations, assessment of local i.v. tolerability, 12-lead electrocardiograms, and laboratory safety tests, including blood chemistry, hematology panels, and urinalysis (urine creatinine and microalbumin) throughout the duration of dosing and continuing through ∼14 days after the last dose. The safety analysis comprised the all-subjects-as-treated (ASaT) population, which included those participants who received ≥1 dose of investigational drug. The number of participants with AEs was descriptively summarized and listed by study part and drug dose. Summary statistics and plots were generated for the change from baseline using the original scale (raw change from baseline) or using the log scale and back-transforming for reporting (percentage of change from baseline).

### Pharmacokinetic parameter analyses.

The target pharmacokinetic parameter for relebactam was determined using an imipenem-relebactam combination tested in a delayed-treatment lung infection model as described previously. The data from the lung infection model suggested that achievement of a mean plasma exposure (area under the curve from 0 h to infinity [AUC_0–∞_]) of >37.5 μM · h (13.1 mg · h/liter) was required for clinical efficacy (35, 36).

Pharmacokinetic parameters were determined from plasma and urine concentration-time data by a noncompartmental approach using Phoenix WinNonlin, version 6.3 (Certara LP, Princeton, NJ, USA). The *C*_EOI_ (concentration at end of infusion) and *T*_max_ (time to maximum concentration) were generated from each analyte's plasma concentration-time data. All AUC values were calculated using the linear trapezoidal method for ascending concentrations and the log trapezoidal method for descending concentrations (calculation method options in WinNonlin). AUC_0–∞_ was calculated as the sum of AUC_0–last_ and *C*_est_, last/λ_*z*_, where AUC_0–last_ is the AUC from time 0 to the time of the last quantifiable concentration, *C*_est_, last is the predicted concentration at the time of the last quantifiable concentration, and λ_*z*_ is the apparent terminal rate constant. For each subject, λ_*z*_ was calculated by regression of the terminal log-linear portion of the plasma concentration-time profile, and the apparent terminal *t*_1/2_ was calculated as the quotient of the natural log of 2 and λ_*z*_. At least three consecutive data points (excluding *C*_max_) in terminal phase were used for λ_*z*_ calculations. Clearance (CL) was calculated as dose/AUC_0–∞_.

The amount of unchanged drug excreted in urine during a collection interval (Ae) was calculated by a cumulative sum of urine (concentration of unchanged drug × volume) per collection interval. The fraction of the dose excreted in urine (fe) was calculated as Ae/dose. Renal clearance (CL_R_) was calculated as Ae/AUC_tau_; where AUC_tau_ is the area under the concentration-versus-time curve from 0 to *t* hour or during the dosing interval at steady state (0 to 24 h for single dose and 0 to 6 h for multiple dose).

## Supplementary Material

Supplemental file 1
