# Supplementary material for: Pharmacokinetics, Safety, and Tolerability of Single and Multiple Doses of Relebactam, a β-Lactamase Inhibitor, in Combination with Imipenem and Cilastatin in Healthy Participants
Source: Antimicrob Agents Chemother. 2018 Aug 27;62(9):e00280-18. doi: 10.1128/AAC.00280-18 (PMC6125551; doi:10.1128/AAC.00280-18)
Supplement: Supplemental file 1 [file zac009187403s1.pdf]

Pharmacokinetics, Safety, and Tolerability of Single and Multiple Doses of Relebactam, a β-Lactamase Inhibitor, in Combination With Imipenem and Cilastatin in Healthy Participants

Running Title (53 of Max 54 characters): Pharmacokinetics of Relebactam and Imipenem/Cilastatin

Elizabeth G. Rhee, MD[a#]; Matthew L. Rizk, PhD[a]; Nicole Calder, PhD[a*]; Marcela Nefliu, PhD[a†]; Steven J. Warrington, MD[b]; Michael S. Schwartz, MS[a]; Eric Mangin, MS[a]; Keith Boundy, MD[a]; Pratik Bhagunde, PhD[a]; Francheska Colon-Gonzalez, PhD[a]; Patricia Jumes, MS, MBA[a]; Yang Liu, PhD[a]; and Joan R. Butterton, MD[a]

[a]Merck & Co., Inc., Kenilworth, NJ, USA

[b]Hammersmith Medicines Research Ltd, London, UK

*GlaxoSmithKline, Uxbridge, Middlesex, UK

†United States Pharmacopeia, Rockville, MD, USA

#Address for Correspondence:

Elizabeth G. Rhee, MD

126 E. Lincoln Ave

Rahway, NJ 07065

Telephone: 732-594-4948

Fax: 732-594-6601

Email: elizabeth.rhee@merck.com

3 Supplemental tables (Max: None listed)

SUPPLEMENTAL MATERIALS

Table S1. Study 1 Summary of Urine Pharmacokinetics Following Administration of Single or Multiple Doses of Relebactam (25 to 1150 mg) With or Without Imipenem/Cilastatin in Healthy Men

Part	Panel	Drug Regimen	N	Day	Ae, mg	fe, %	CL_R, mL/min
**Relebactam, Mean ± SD**							
I	A	25 mg Relebactam	6	1	24.1±1.31	96.5±5.25	177±29.4
I	A	50 mg Relebactam	6	1	48.3±2.22	96.7±4.45	166±21.8
I	A	125 mg Relebactam	6	1	128±4.19	103±3.35	159±19.6
I	A	250 mg Relebactam	6	1	246±6.58	98.4±2.63	153±16.5
I	A	50 mg Relebactam + 500 mg Imipenem/Cilastatin	6	1	55.1±14.3	110±28.5	182±56.5
I	B	250 mg Relebactam	6	1	249±22.9	99.6±9.18	144±11.0
I	B	500 mg Relebactam	6	1	474±15.6	94.7±3.11	129±20.7
I	B	1000 mg Relebactam	6	1	972±29.8	97.2±2.98	127±20.2
I	B	1150 mg Relebactam	6	1	1110±57.5	96.8±5.00	137±20.6
I	B	500 mg Relebactam + 500 mg Imipenem/Cilastatin	6	1	541±107	108±21.3	146±45.7
II	C	50 mg Relebactam + 500 mg Imipenem/Cilastatin QID x 7	5	7	44.6±7.41	89.2±14.8	141±31.8
II	D	125 mg Relebactam + 500 mg Imipenem/Cilastatin QID x 7	6	7	124±4.63	99.3±3.71	138±20.4
II	E	125 mg Relebactam + 500 mg Imipenem/Cilastatin QID x 7	6	7	124±3.57	99.5±2.85	143±14.6
II	F	250 mg Relebactam + 500 mg Imipenem/Cilastatin QID x 7	6	7	227±6.69	90.8±2.67	135±24.2
III	G	375 mg Relebactam + 500 mg Imipenem/Cilastatin QID x 7	6	7	357±11.0	95.2±2.92	144±23.8
III	H	500 mg Relebactam + 500 mg Imipenem/Cilastatin QID x 7	6	7	498±23.1	99.5±4.62	144±19.3
III	I	625 mg Relebactam + 500 mg Imipenem/Cilastatin QID x 7	6	7	607±31.6	97.2±5.06	151±23.3
**Imipenem, Mean ± SD**							
I	A	500 mg Imipenem/Cilastatin	8	1	263±52.1	52.6±10.4	106±20.3
I	B	500 mg Imipenem/Cilastatin	8	1	287±58.5	57.3±11.7	114±19.4
I	A	50 mg Relebactam + 500 mg Imipenem/Cilastatin	6	1	318±102	63.6±20.5	131±46.9
I	B	500 mg Relebactam + 500 mg Imipenem/Cilastatin	6	1	340±78.4	68.1±15.7	130±39.7
II	C	50 mg Relebactam + 500 mg Imipenem/Cilastatin QID x 7	5	7	314±86.0	62.7±17.2	137±28.7
II	D	125 mg Relebactam + 500 mg Imipenem/Cilastatin QID x 7	6	7	312±45.9	62.3±9.17	126±28.9
II	E	125 mg Relebactam + 500 mg Imipenem/Cilastatin QID x 7	6	7	327±16.1	65.3±3.21	139±15.0
II	F	250 mg Relebactam + 500 mg Imipenem/Cilastatin QID x 7	6	7	314±29.1	62.7±5.82	128±27.1
III	G	375 mg Relebactam + 500 mg Imipenem/Cilastatin QID x 7	6	7	342±16.2	68.5±3.24	134±16.3
III	H	500 mg Relebactam + 500 mg Imipenem/Cilastatin QID x 7	6	7	353±25.8	70.6±5.16	133±21.8
III	I	625 mg Relebactam + 500 mg Imipenem/Cilastatin QID x 7	6	7	327±35.1	65.5±7.03	139±22.5
**Cilastatin, Mean ± SD**							
I	A	500 mg Imipenem/Cilastatin	8	1	417±84.3	83.3±16.9	173±42.1
I	B	500 mg Imipenem/Cilastatin	8	1	468±33.5	93.7±6.70	173±40.4
I	A	50 mg Relebactam + 500 mg Imipenem/Cilastatin	6	1	475±125	95.0±25.0	202±64.9
I	B	500 mg Relebactam + 500 mg Imipenem/Cilastatin	6	1	463±109	92.6±21.9	184±81.1
II	C	50 mg Relebactam + 500 mg Imipenem/Cilastatin QID x 7	5	7	352±89.8	70.4±18.0	155±57.0
II	D	125 mg Relebactam + 500 mg Imipenem/Cilastatin QID x 7	6	7	426±21.5	85.2±4.30	188±31.7
II	E	125 mg Relebactam + 500 mg Imipenem/Cilastatin QID x 7	6	7	400±28.1	79.9±5.61	193±40.1
II	F	250 mg Relebactam + 500 mg Imipenem/Cilastatin QID x 7	6	7	383±19.5	76.6±3.91	184±30.2
III	G	375 mg Relebactam + 500 mg Imipenem/Cilastatin QID x 7	6	7	416±35.8	83.1±7.16	180±38.6
III	H	500 mg Relebactam + 500 mg Imipenem/Cilastatin QID x 7	6	7	409±31.2	81.9±6.24	156±33.1
III	I	625 mg Relebactam + 500 mg Imipenem/Cilastatin QID x 7	6	7	417±38.4	83.4±7.68	177±35.8

Ae, amount of drug excreted unchanged in urine; CLR, renal clearance; fe, fraction excreted; SD, standard deviation.

Table S2. Study 1 Plasma Pharmacokinetic Parameters Following Administration of Multiple Doses of Relebactam (50-625 mg) With 500 mg Imipenem/Cilastatin in Healthy Men Over 7 Days (Part II and Part III) and 14 Days (Part IV)

Relebactam dose + 500 mg Imipenem/Cilastatin	Part	Panel	Day	N	$AUC_{0\text{-}6}$, μM · h	C_{EOI}, μM	Terminal $t_{1/2}$[a], h	V_z	V_{ss}, L	CL, mL/min
**Relebactam, Mean ± SD**										
50 mg	II	C	1	6	17.6±1.94	9.84±1.03	1.64±0.084	—	—	—
50 mg	II	C	7	5	15.4±2.10	9.11±0.302	1.42±0.122	19.3±1.58	18.3±1.35	158±22.0
125 mg	II	D	1	6	44.0±4.86	26.6±3.99	1.44±0.208			
125 mg	II	D	7	6	43.7±5.49	27.5±4.76	1.43±0.186	17.3±2.22	14.9±2.82	139±16.4
125 mg	II	E	1	6	42.5±5.29	24.7±2.80	1.42±0.213	—	—	—
125 mg	II	E	7	6	41.9±4.47	26.0±3.45	1.34±0.170	16.9±1.32	14.9±1.45	144±14.7
250 mg	II	F	1	6	78.9±11.5	47.3±9.10	1.63±0.130	—	—	—
250 mg	II	F	7	6	82.7±13.8	49.4±11.0	1.65±0.224	21.2±3.00	17.6±3.01	148±14.7
375 mg	III	G	1	6	105±14.8	59.3±11.5	1.48±0.147	—	—	—
375 mg	III	G	7	6	121±16.2	70.2±10.2	1.85±0.241	24.6±5.29	18.2±2.92	151±22.9
500 mg	III	H	1	6	162±20.8	98.4±23.4	1.54±0.232	—	—	—
500 mg	III	H	7	6	168±20.2	103±20.6	1.68±0.193	21.4±4.49	16.7±3.61	144±17.3
625 mg	III	I	1	6	187±23.7	119±24.1	1.47±0.186	—	—	—
625 mg	III	I	7	6	196±28.7	117±24.4	1.73±0.180	23.3±3.51	18.2±3.17	155±22.8
500 mg	IV	J	1	12	145±11.9	88.3±14.4	1.58±0.167	—	—	—
500 mg	IV	J	14	11	152±11.3	93.0±8.25	1.74±0.218	24.3±4.17	18.8±1.75	158±11.6
500 mg	IV	K	1	12	154±20.3	97.9±14.4	1.46±0.102	—	—	—
500 mg	IV	K	14	11	151±21.5	97.8±16.4	1.69±0.190	24.0±4.92	18.0±2.95	161±24.0
**Imipenem, Mean ± SD**										
50 mg	II	C	1	6	140±20.8	103±8.11	1.13±0.093	—	—	—
50 mg	II	C	7	5	127±16.3	96.7±6.62	1.09±0.065	21.0±2.99	16.5±1.45	222±27.8
125 mg	II	D	1	6	138±15.3	107±17.7	1.07±0.125	—	—	—
125 mg	II	D	7	6	141±19.6	113±21.2	1.11±0.090	19.2±2.90	15.1±3.31	200±24.8
125 mg	II	E	1	6	140±18.3	106±12.3	1.05±0.128	—	—	—
125 mg	II	E	7	6	132±9.02	105±12.1	1.01±0.064	18.6±1.69	15.3±1.41	212±14.9
250 mg	II	F	1	6	142±18.2	110±22.0	1.15±0.093	—	—	—
250 mg	II	F	7	6	140±23.1	109±25.9	1.13±0.126	20.1±3.87	16.1±3.77	204±38.1
375 mg	III	G	1	6	137±17.7	96.8±17.9	1.06±0.073	—	—	—
375 mg	III	G	7	6	145±18.3	112±14.3	1.17±0.154	20.3±4.87	15.3±2.42	195±25.9
500 mg	III	H	1	6	153±22.1	118±27.8	1.09±0.115	—	—	—
500 mg	III	H	7	6	150±22.6	119±26.9	1.04±0.039	17.0±2.75	14.1±3.44	189±28.8
625 mg	III	I	1	6	135±14.5	110±21.4	1.09±0.106	—	—	—
625 mg	III	I	7	6	132±16.6	101±19.7	1.05±0.111	19.5±2.84	16.4±3.04	213±26.9
0 mg	III	I	1	1	189	137	1.11			
0 mg	III	I	7	1	202	142	1.17	13.6	10.9	135
500 mg	IV	J	1	12	122±9.27	94.5±14.6	1.09±0.105	—	—	—
500 mg	IV	J	14	11	132±11.5	106±11.1	1.10±0.083	20.3±1.58	16.1±1.68	212±19.5
500 mg	IV	K	1	12	145±21.8	116±17.2	1.05±0.077	—	—	—
500 mg	IV	K	14	11	138±18.8	115±18.7	1.06±0.095	19.0±3.37	14.8±2.30	205±29.4
0 mg	IV	J	1	4	131±17.3	106±19.7	1.08±0.071	—	—	—
0 mg	IV	J	14	3	156±5.32	118±3.96	1.19±0.089	17.9±0.07	14.1±0.051	174±6.90
0 mg	IV	K	1	4	144±17.1	102±13.0	1.07±0.101	—	—	—
0 mg	IV	K	14	4	154±14.7	124±29.9	1.11±0.150	17.2±2.47	13.8±2.49	178±16.1
**Cilastatin, Mean ± SD**										
50 mg	II	C	1	6	127±24.8	106±12.0	1.01±0.142	—	—	—
50 mg	II	C	7	5	110±22.0	97.5±9.53	0.895±0.134	16.9±2.07	13.0±0.847	218±43.5

125 mg	II	D	1	6	120±13.0	107±16.7	0.914±0.161	—	—	—
125 mg	II	D	7	6	108±15.8	103±14.8	0.942±0.161	18.1±2.83	12.6±2.89	220±29.5
125 mg	II	E	1	6	111±15.4	103±12.2	0.860±0.245	—	—	—
125 mg	II	E	7	6	98.7±14.5	98.8±8.27	0.765±0.147	16.0±1.67	12.2±1.05	240±38.2
250 mg	II	F	1	6	110±14.2	103±20.0	0.998±0.087	—	—	—
250 mg	II	F	7	6	99.1±16.2	98.3±19.9	0.996±0.132	20.7±2.73	14.0±2.73	240±42.1
375 mg	III	G	1	6	114±12.8	95.4±14.6	0.900±0.093	—	—	—
375 mg	III	G	7	6	110±17.1	102±11.6	0.983±0.197	19.2±6.07	13.1±2.13	215±32.3
500 mg	III	H	1	6	136±22.5	115±21.9	1.01±0.146	—	—	—
500 mg	III	H	7	6	125±21.8	113±22.2	0.891±0.108	14.9±3.10	11.5±2.83	191±33.6
625 mg	III	I	1	6	126±12.4	111±17.4	0.960±0.142	—	—	—
625 mg	III	I	7	6	111±14.2	99.5±15.5	0.949±0.163	17.7±3.36	13.3±2.49	212±28.7
0 mg	III	I	1	1	167	144	0.962	—	—	—
0 mg	III	I	7	1	169	146	0.963	11.3	8.48	136
500 mg	IV	J	1	12	106±17.2	98.5±14.6	0.874±0.117	—	—	—
500 mg	IV	J	14	11	99.3±12.1	97.6±11.1	0.956±0.125	19.8±2.88	13.4±1.48	237±29.7
500 mg	IV	K	1	12	116±19.0	111±17.0	0.843±0.133	—	—	—
500 mg	IV	K	14	11	102±18.3	104±17.0	0.930±0.258	20.5±7.58	12.5±1.75	234±38.5
0 mg	IV	J	1	4	110±7.17	107±6.53	0.862±0.088	—	—	—
0 mg	IV	J	14	3	115±3.13	110±1.87	0.995±0.050	17.3±1.28	11.8±0.45	201±5.32
0 mg	IV	K	1	4	126±37.9	104±10.4	0.908±0.182	—	—	—
0 mg	IV	K	14	4	122±31.7	116±29.5	0.918±0.186	16.2±5.55	11.8±3.21	197±44.7

[a]Harmonic mean ± pseudo SD.

AUC, area under the concentration-time curve; C_{EOI}, concentration at end of infusion; CL, clearance; SD, standard deviation; $t_{1/2}$, apparent terminal half-life; T_{max}, Time of maximum concentration; V_z, volume of distribution; V_{ss}, Volume of distribution at steady state.

Table S3. Study 1 Participants With Specific Drug-Related Adverse Events Following Single-Dose Administration in Healthy Men

	Relebactam							Relebactam + Imipenem/Cilastatin					Total, N[a]
**Relebactam dose, mg**	**25**	**50**	**125**	**250**	**500**	**1000**	**1150**	**0**	**50**	**500**	**0**	**0**	
**Imipenem/Cilastatin dose, mg**	**0**	**0**	**0**	**0**	**0**	**0**	**0**	**500**	**500**	**500**	**500**	**500**	
**n[a]**	**6**	**6**	**6**	**12**	**6**	**6**	**6**	**16**	**6**	**6**	**4**	**16**	**16**
**Drug-related AE, n (%)[b]**													
**General disorders and administration site conditions**	**0**	**0**	**0**	**1 (8.3)**	**0**	**0**	**1 (16.7)**	**0**	**0**	**0**	**0**	**0**	**2 (12.5)**
Feeling of body temperature change	0	0	0	0	0	0	1 (16.7)	0	0	0	0	0	1 (6.3)
Infusion site erythema	0	0	0	1 (8.3)	0	0	0	0	0	0	0	0	1 (6.3)
**Infections and infestations**	**0**	**0**	**0**	**0**	**0**	**0**	**0**	**0**	**0**	**0**	**0**	**1 (6.3)**	**1 (6.3)**
Rhinitis	0	0	0	0	0	0	0	0	0	0	0	1 (6.3)	1 (6.3)
**Nervous system disorders**	**0**	**0**	**1 (16.7)**	**2 (16.7)**	**2 (33.3)**	**1 (16.7)**	**0**	**1 (6.3)**	**0**	**0**	**1 (25)**	**2 (12.5)**	**6 (37.5)**
Dizziness	0	0	0	0	0	0	0	0	0	0	0	1 (6.3)	1 (6.3)
Headache	0	0	0	1 (8.3)	0	0	0	1 (6.3)	0	0	0	1 (6.3)	2 (12.5)
Paresthesia	0	0	1 (16.7)	0	0	1 (16.7)	0	0	0	0	0	0	2 (12.5)
Somnolence	0	0	0	1 (8.3)	2 (33.3)	0	0	0	0	0	1 (25)	0	3 (18.8)
**Psychiatric disorders**	**0**	**0**	**0**	**0**	**0**	**1 (16.7)**	**0**	**0**	**0**	**0**	**0**	**0**	**1 (6.3)**
Elevated mood	0	0	0	0	0	1 (16.7)	0	0	0	0	0	0	1 (6.3)
Restlessness	0	0	0	0	0	1 (16.7)	0	0	0	0	0	0	1 (6.3)

[a]n values reflect total number of patients administered a particular dose; N reflects the total number of patients in the particular cohort.

[b]Every participant was counted a single time for each adverse event category, so total values in the total AE categories may be less than the sum of the individual AEs.

AE, adverse event.
